# Fatty Acid Content and Composition of the Morphological Fractions of *Cistus Ladanifer* L. and Its Seasonal Variation

**DOI:** 10.3390/molecules25071550

**Published:** 2020-03-28

**Authors:** Eliana Jerónimo, Liliana Cachucho, David Soldado, Olinda Guerreiro, Rui J. B. Bessa, Susana P. Alves

**Affiliations:** 1 Centro de Biotecnologia Agrícola e Agro-Alimentar do Alentejo (CEBAL)/Instituto Politécnico de Beja (IPBeja), 7801-908 Beja, Portugal; liliana.cachucho@cebal.pt (L.C.); david.soldado@cebal.pt (D.S.); olinda.guerreiro@cebal.pt (O.G.); 2 MED—Mediterranean Institute for Agriculture, Environment and Development, CEBAL, 7801-908 Beja, Portugal; 3 CIISA—Centro de Investigação Interdisciplinar em Sanidade Animal, Faculdade de Medicina Veterinária, Universidade de Lisboa, Avenida da Universidade Técnica, 1300-477 Lisboa, Portugal; rjbbessa@fmv.ulisboa.pt (R.J.B.B.); susanaalves@fmv.ulisboa.pt (S.P.A.)

**Keywords:** *Cistus ladanifer* L., plant morphological fractions, seasonal variation, fatty acids, branched-chain fatty acids

## Abstract

*Cistus ladanifer* L. is a shrub from Cistaceae family, widespread in Mediterranean countries. Fatty acids (FA) have multiple roles in plants and are involved in adaption mechanisms to environmental conditions. This work evaluated the FA content and composition of each morphological fraction of *C. ladanifer* (leaves, stems, flower buds, flowers and seed heads) throughout a full year. *Cistus ladanifer* plants were collected in southern Portugal, during four consecutive seasons (18 plants/season), and the different morphological plant fractions (leaves, stems, flower buds, flowers and seed heads) were separated. *Cistus ladanifer* morphological fractions showed distinct FA compositions, being possible to discriminate three groups—the leaves that showed to be dominated by saturated FA (main 20:0) and contain branched-chain FA (iso-19:0 and iso-21:0); the stems that are composed mainly by SFA (main 22:0); and the reproductive organs that showed higher contents of polyunsaturated FA (PUFA) and the 16:0 as the main SFA. The FA composition of leaves changed over seasons, with replacement of the PUFA by monounsaturated FA and branched-chain FA during hot seasons. Regarding the other *C. ladanifer* morphological fractions, the FA composition was more stable over seasons, suggesting that leaves are more prone to adaptations to environmental changes.

## 1. Introduction

*Cistus ladanifer* L., commonly known as rockrose, is a perennial shrub that is distributed from southern France to the north of Morocco and Algeria, being particularly abundant in the southwestern region of Iberian Peninsula [1]. *Cistus ladanifer* is highly resistant to drought, and well adapted to climatic conditions of Mediterranean countries, where it grows spontaneously principally in forest areas and uncultivated lands.

*Cistus ladanifer* has been used since ancient times in traditional medicine, and over time diverse biological activities have been identified in *C. ladanifer* extracts, as antioxidant [2,3,4,5,6], antimicrobial [7,8,9,10], antihypertensive [11], anti-inflammatory and analgesic [12] effects, and inhibition of the proliferation of pancreatic and breast cancer cells [10]. Despite the recognized biological activities, *C. ladanifer* has only been valued by the perfume and cosmetics industry. In the last decade, a great effort has been developed to increase the *C. ladanifer* uses, including its application in ruminant nutrition [13,14,15,16] and for bioethanol production [17]. 

Due to its main use in the perfume and cosmetics industry, greater attention has been given to the chemical composition of *C. ladanifer* essential oil [18,19,20] and labdanum exudate [21,22,23,24]. As far as we know, the proximate composition of the aerial parts of *C. ladanifer* was only evaluated in two studies [6,25]. The phytochemical composition of the *C. ladanifer* aerial parts has also been explored, with focus on the polyphenolic compounds, vitamins and fatty acids (FA) [2,10]. 

The FA composition of *C. ladanifer* aerial parts (blend of stems, leaves and reproductive organs) throughout a full year was characterized by our team [26]. More than 70% of the total FA in *C. ladanifer* aerial parts are saturated fatty acids (SFA), composed mainly by 20:0 and 16:0. The oleic (18:1 *cis*-9), linoleic (18:2n-6) and α-linolenic (18:3n-3) acids were the only unsaturated FA found in *C. ladanifer* aerial parts. The presence of two odd mono-methyl branched-chain fatty acids (BCFA), i.e., the iso-19:0 and iso-21:0, were also identified in the aerial parts of *C. ladanifer*. Despite this previous study, it is not known which aerial part of *C. ladanifer* contains the highest content of SFA and if the BCFA are present in all plant morphologic fractions. Branched-chain FA present bioactivities such as antitumoral effect [27,28] and reduction of the incidence of necrotizing enterocolitis [27,29], which increase the interest on this FA group. So, in addition to already known bioactive compounds, *C. ladanifer* may also be a source of odd BCFA. 

We have previously suggested that FA of *C. ladanifer* aerial parts may play an important role in plant adaptation mechanisms to environmental temperatures, with the replacement of polyunsaturated fatty acids (PUFA) by BCFA at high temperatures [26]. However, in that work the FA composition was analyzed in a blend of several morphological fractions of aerial part of *C. ladanifer* (i.e., mixture of stems, leaves and reproductive organs), thus there was no information about the variations in FA composition in each morphological fraction during the plant growth cycle, as well as in response to seasonal environmental changes. The Mediterranean environments are characterized by long-lasting supra-optimal temperatures and light, thus the knowledge about the FA composition of each morphological fraction according to seasonal variations may help to understand the *C. ladanifer* adaptation mechanisms to environmental conditions. So, the present work aims to characterize the morphological fractions of *C. ladanifer* in terms of FA composition, specifically leaves, stems, flower buds, flowers and seed heads and to evaluate possible FA composition changes throughout a full year. 

## 2. Results

### 2.1. Total Fatty Acid Content and Composition of the Morphological Fractions of Cistus Ladanifer

**Figure 1:**
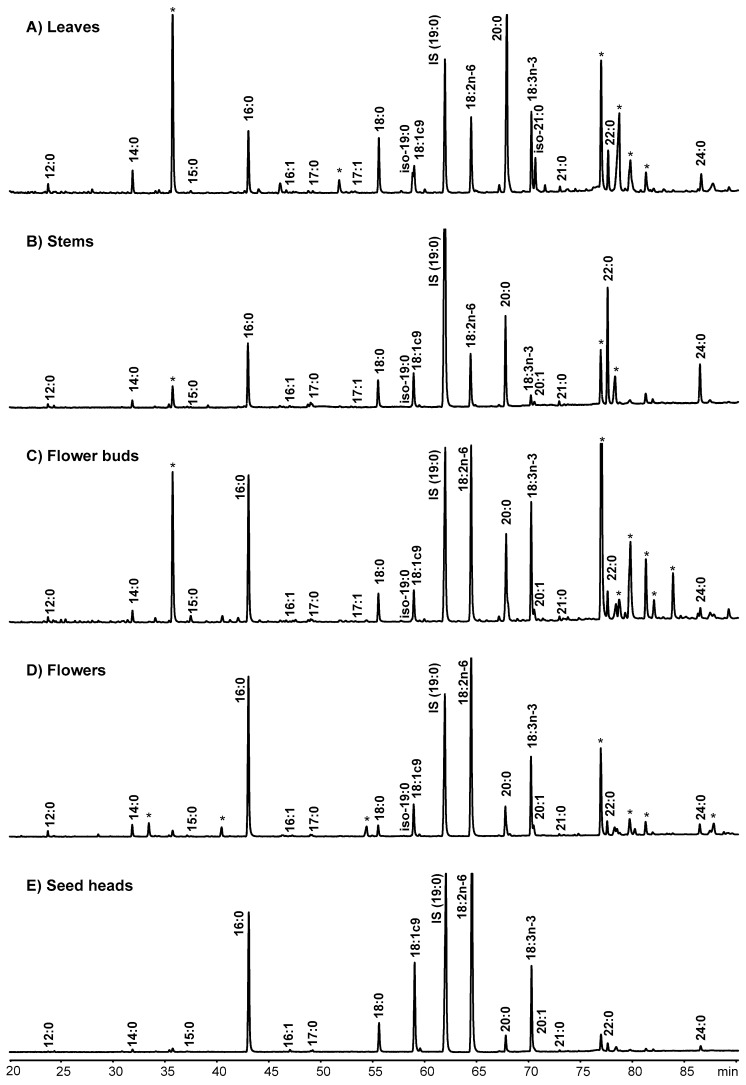
Partial gas-liquid chromatogram of the fatty acid methyl esters using a SP-2560 capillary column. (**A**) *Cistus ladanifer* leaves; (**B**) *C. ladanifer* stems; (**C**) *C. ladanifer* flower buds; (**D**) *C. ladanifer* flowers; (**E**) *C. ladanifer* seed heads (*, non-fatty acid compounds).

The FA content and composition of *C. ladanifer* leaves, stems, flower buds and flowers and seed heads are presented in Table 1, Table 2, Table 3 and Table 4, respectively. The gas-liquid chromatograms of the various morphological fractions are presented in Figure 1 and it can be observed that several non-FA compounds were present in some of the *C. ladanifer* morphological fractions. The identification and presence of some of those compounds was already discussed previously [26] and will not be the focus of this work. Although it is not intended to make a comparison among the various morphological fractions, but the characterization of each of them, it is evident differences in the FA content and composition of *C. ladanifer* morphological fractions. The total FA content of leaves ranged from 13.6 to 17.5 mg/g DM. Stems had the lowest FA content, averaging 3.46 mg/g DM. Flower buds and flowers showed 11.9 and 14.6 mg/g DM of total FA, respectively. The total FA content of seed heads showed great variation among seasons, ranging between 9.70 in winter and 22.7 mg/g DM in summer.

The FA composition of *C. ladanifer* leaves and stems is dominated by SFA, comprising 63% and 76% of total FA, respectively. Polyunsaturated fatty acids are the second most abundant FA in these morphological fractions, representing 27% and 15% of total FA in leaves and stems, respectively. In flowers and seed heads, SFA represented, respectively, 42 and 29% of total FA, while PUFA are dominants, reaching 50 and 58% of total FA, respectively. Regarding the flower buds, SFA and PUFA levels were more equilibrated, with 46% of SFA and 44% of PUFA in total FA. Monounsaturated fatty acids (MUFA) represented 5.4%, 9.6%, 7.0%, 7.8% and 13% of total FA in leaves, stems, flower buds, flowers and seed heads, respectively. In all morphological fractions were identified 10 SFA, specifically 12:0, 14:0, 15:0, 16:0, 17:0, 18:0, 20:0, 21:0, 22:0 and 24:0. Regarding to unsaturated FA, were identified two MUFA (18:1 *cis*-9 and 20:1) and two PUFA (18:2n-6 and 18:3n-3) in all morphological fractions, with exception of 20:1 that was not found in leaves. Two BCFA, iso-19:0 and iso-21:0, were identified in leaves, representing 5.2% of total FA. In stems, flower buds and flowers were only identified the iso-19:0, but in residual levels (0.13, 0.27 and 0.15% of total FA in stems, flower buds and flowers, respectively), while in seed heads no BCFA were found. 

The main FA present in leaves were the 20:0, 16:0, 18:3n-3 and 18:2n-6, being the 20:0 the most abundant FA (ranged from 25% to 34% of total FA). The proportion of 18:3n-3 ranged from 11.5 to 21% of total FA. The 16:0 and 18:2n-6 comprised 13.1 and 10.7% of total FA, respectively. Regarding the BCFA in leaves, the iso-19:0 ranged from 1.50 and 2.90% of total FA and iso-21:0 from 2.66 and 4.12% of total FA. 

The 22:0 represented the most abundant FA in stems (ranged from 21% to 27% of total FA), followed by 20:0 and 16:0 that showed similar levels (17.3% and 16.5% of total FA, respectively), and then 18:2n-6 (12%) and 18:1 *cis*-9 (ranged from 6.8% to 11.2% of total FA).

Either in flower buds as in flowers, the main FA was 18:2n-6 (31% and 37% of total FA, respectively), followed by 16:0 (24 and 28% of total FA, respectively). The 18:3n-3, represented about 13% of total FA in both morphological fractions, and the proportion of 20:0 comprised 12% and 6% of total FA in flower buds and in flowers, respectively.

In seed heads, 4 individual FA, 18:2n-6, 16:0, 18:3n-3 and 18:1 *cis*-9, comprised more than 90% of total FA. The 18:2n-6 was the most abundant FA in seed heads (46% of total FA), followed by 16:0 (21% of total FA). The 18:3n-3 and the 18:1 *cis*-9 represented each of them about 12% of total FA. 

**Figure 2:**
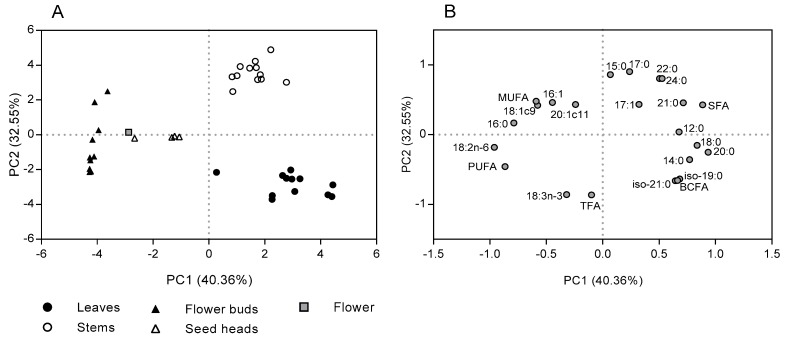
Scores (**A**) and loadings (**B**) of the two first principal components (PC) computed using the fatty acid content and composition of *C. ladanifer* morphological fractions.

Results of principal component analysis (PCA) showed that the first two factors explain 72.9% (Figure 2) of the variance, allowing the discrimination of the morphological fractions in function of their FA content and composition. The first factor (PC 1) accounted for the 40.4% of the variability, relating positively with total and individual SFA (except 16:0) and total and individual BCFA, and negatively with 16:0, total and individual MUFA (except 17:1), total PUFA and 18:2n-6. The second factor (PC 2) explained 32.6% of the variability of the data set, relating positively with several SFA (15:0, 17:0, 22:0 and 24:0) and negatively with total FA and 18:3n-3. As shown on the PCA (Figure 2A), it was possible to identify the presence of three distinct groups, the first composed by the stems, the second composed by the leaves, and the last group by the flower buds, flowers and seed heads. 

### 2.2. Seasonal Variation in the Fatty Acid Content and Composition in the Morphological Fractions of Cistus Ladanifer

The total FA content of leaves was affected by season (*p* = 0.037), with higher levels during winter and autumn (17.25 mg/g DM) than in spring and summer (13.6 mg/g DM). In stems and flower buds, the total FA content did not vary throughout seasons (*p* = 0.818 and *p* = 0.900, respectively). The total FA content of seed heads tended to change over the year (*p* = 0.069), with higher values during summer and autumn (22.5 mg/g DM) than in winter (9.70 mg/g DM), while in spring the average value was similar to the other seasons.

The FA composition of leaves was more variable throughout seasons than in other morphological fractions. Indeed, all FA sums, as well as seven individual FA of leaves were affected by season. In summer was found the lower content of 14:0 (0.29 mg/g DM) and the higher content of 18:1 *cis*-9 (1.36 mg/g DM) compared with the other seasons (0.61 and 0.59 mg/g DM of 14:0 and 18:1 *cis*-9, respectively). The content of 18:0 remained constant during winter, spring and summer (1.11 mg/g DM) but increased in autumn (1.73 mg/g DM). The content of 20:0 decreased (*p* = 0.003) between winter and spring and then increased during summer and autumn, reaching similar values to winter. The 18:3n-3 decreased between winter (3.62 mg/g DM) and summer (1.34 mg/g DM), remaining constant until autumn.

In leaves, the sum of BCFA and individual BCFA were affected by season (*p* < 0.05). The sum of BCFA and iso-19:0 were higher in autumn (1.23 and 0.50 mg/g DM, respectively) compared with the other seasons (0.68 and 0.26 mg/g DM, respectively). Higher levels of iso-21:0 were also found in autumn comparatively to winter and spring (0.73 and 0.38 mg/g DM, respectively). Total n-SFA was higher during winter and autumn (11.2 mg/g DM) than during spring and summer (8.28 mg/g DM). The content of PUFA decreased from winter (5.29 mg/g DM) to summer, remaining constant until autumn (3.48 mg/g DM).

**Figure 3:**
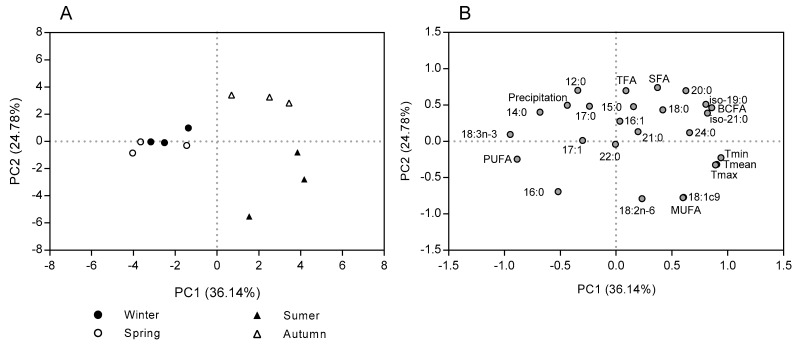
Scores (**A**) and loadings (**B**) of the two first PCs computed using the fatty acid content and composition of leaves, as well as data about temperature and precipitation among seasons.

Principal component analysis of leaves data, explains 60.9% of the variability, allowing the discrimination of leaves in the function of their FA content and composition, environment temperatures and precipitation (Figure 3). As shown in Figure 3A, it is possible to identify three distinct groups, the first composed by the leaves collected during autumn, the second composed by the leaves from summer, and the last group composed of the leaves collected during spring and winter. The first factor (PC 1) accounted for 36.1% of the variability, relating positively with total and individual BCFA, and temperatures (mean, maximum and minimum) and negatively with total PUFA and 18:3n-3. The second factor (PC 2) accounted for 24.8% of the variability, relating positively with total FA, total SFA, most SFA and precipitation, and negatively with total MUFA, 18:1 *cis*-9, 18:2n-6 and 16:0 (Figure 3B). 

In stems only 3 FA varied throughout seasons, the 12:0 (*p* = 0.008), 18:1 *cis*-9 (*p* = 0.038) and 18:3n-3 (*p* = 0.011). The 12:0 remained unchanged during winter and spring (0.012 mg/g DM), increasing in summer and autumn (0.021 mg/g DM). Conversely, 18:3n-3 that also remained unchanged during winter and spring (0.12 mg/g DM), decreased in summer and autumn (0.07 mg/g DM). The 18:1 *cis*-9 increased from winter to summer (0.25 vs 0.42 mg/g DM), decreasing in autumn to similar levels found in winter.

The FA composition of *C. ladanifer* flower buds, which was only present in winter and spring, did not vary between the two seasons. Only a tendency (*p* = 0.069) of 18:1 *cis*-9 to increase from winter (0.55 mg/g DM) to spring (1.04 mg/g DM) was observed. In *C. ladanifer* seed heads were only observed trends regarding to the changes of the FA composition throughout seasons, with a progressive increase of the 16:0, 18:2n-6, 18:3n-3 and sum of PUFA from winter to summer, remaining unchanged until autumn. 

## 3. Discussion

*Cistus ladanifer* is a perennial shrub with 1–2 m of height [30], with branches of very rigid and lignified wood covered by a sticky and viscous bark and lanceolate green leaves presented in a decussate arrangement and welded at the base [1]. Although the time and duration of each development stage of plant depends on both the location and the climate conditions, it is consensual that vegetative growth of *C. ladanifer* starts after the first autumn rains, being reduced during the summer dry season [30,31]. The *C. ladanifer* plant used in the present work was composed mainly of stems and leaves, ranging from 679 to 750 g/kg DM of stems and from 214 to 276 g/kg DM of leaves in whole plants (data not shown). According to our results, leaves showed the higher FA content compared to stems, and thus leaves are an important contribute to the total FA content of the *C. ladanifer* aerial parts, which ranged from 5.4 to 8.6 mg/g of total FA [26]. 

Regarding the FA composition of both leaves and stems, it was found to be similar to the *C. ladanifer* aerial parts [26], with a high proportion of SFA. In the aerial part of *C. ladanifer*, SFA comprised more than 70% of total FA, while the PUFA proportion ranged from 4.2 and 22% of total FA [26]. In accordance with the previous results on aerial parts of *C. ladanifer*, the main SFA present in leaves were also the 20:0 and 16:0. Conversely, in the stems, the predominant SFA was the 22:0, which represents only 10–13% of total SFA with linear chain in aerial parts of *C. ladanifer* [26]. The most interesting result was the almost exclusive presence of BCFA in leaves. The presence of iso BCFA in aerial parts of *C. ladanifer* was reported for the first time by Guerreiro et al. [26]. Branched-chain FA are mostly derived from bacteria and thus constitute a major component of the membranes of several bacteria [32]. Branched-chain FA are also found in appreciable levels in ruminant meat and milk and in minor levels in fermented foods such as sauerkraut and miso [33]. In plant lipids, BCFA have been rarely found, as reviewed by Eibler et al. [34]. 

In the other morphological fractions, the PUFA gained importance. Flower buds and flowers showed more equal levels of SFA and PUFA, while in seed heads the FA composition is dominated by PUFA. At the end of winter, flower buds formation begins and, although some flowers may arise at the end of winter, the flowering occurs mainly during spring [6,31]. *Cistus ladanifer* presents ephemeral flowers (1–3 days) [1] with 5–100 mm of diameter and composed by five white petals with a maroon spot at the base [31]. Regarding to the pollen production, Talavera et al. [31] reported values between 494,000 to 782,000 grains per flower. The fat content of the honeybee-collected pollen in Spain, classified as monofloral *Cistus* pollen, ranged from 4.80 to 7.18 g/100 g and the FA profile is characterized by the high proportion of unsaturated FA (about 66%), mainly composed by 18:2n-6, 18:3n-3 and 18:1 *cis*-9, while 16:0 is the most abundant SFA [35]. In the lipophilic fraction of honeybee pollen from *C. ladanifer*, the most abundant FA are the 18:2n-6, 18:3n-3, 18:1 *cis*-9 and 16:0 [36]. The major FA in *C. ladanifer* flower buds and flowers are also 18:2n-6, 18:3n-3, 18:1 *cis*-9 and 16:0, being the 18:2n-6 the most abundant, which is consistent with the FA profile of honeybee [35,36]. 

*Cistus ladanifer* seed heads are a globular and lignified structure with 6–12 valves, and each seed head produces large number of small (0.5–1 mm and about 0.27 mg) polyhedral seeds (aprox. 250 per valve) [37,38,39]. An adult plant of *C. ladanifer* can produce up to 158,000 seeds per year [39], which are released over a long period of time (8–10 months), starting in the middle of summer [39]. In the present work seed heads were found practically all year round, only in April were not identified seed heads in plants. After flowering, the ovary/young fruit is completely enclosed by the sepals and during fruit maturation, the leaves and bracts abscise and pedicels elongate and lignify [31]. In early summer the fruits are mature and the seed heads are exposed [31]. During the summer, seed heads begin to open, and with the early wind and rain of autumn, there is seed dispersal [31]. So, during winter and spring, the seed heads present in plants resulted from the previously reproductive cycle, with incomplete seed heads and many valves open and without seeds. On the other hand, new seed heads are found in plants during summer and autumn, many of them still closed. Thus, it is expected that from winter to spring the seed quantity into seed heads will be lower than in the summer and autumn, which can explain the lower FA content observed during winter and spring than in the other seasons.

Although the FA composition of *C. ladanifer* seed was not analyzed, and the seed heads fraction containing both the external structure and the seeds, the FA profile observed in seed heads is in agreement with the FA composition of several seeds from the Cistaceae family [40]. The FA profile of *Cistus* seeds is similar to seeds from sunflower, soybean, corn, cotton or rape, and are characterized by high proportions of PUFA, mainly 18:2n-6 [41]. Moreover, the 16:0 was the most predominant SFA found in seed heads, which is in agreement with the FA profile of soybean, corn and cotton seeds [41]. 

The distinct FA composition among various morphological fractions is corroborated by the PCA, allowing the identification of three distinct groups (Figure 2A). Although the FA composition of leaves and stems are dominated by SFA with linear chain, the distinct composition of some individual FA and particularly the almost exclusive presence of iso BCFA in leaves contributed to the discrimination between stems and leaves. The main SFA present in stems (22:0) showed a higher association with this morphological fraction, while leaves had great association with total and individual BCFA and 20:0. The other morphological fractions of *C. ladanifer*, corresponding to reproductive organs, had a higher association with PUFA and MUFA, as well as with 16:0. Curiously, the 16:0 was the major SFA present in all reproductive organs. 

**Figure 4:**
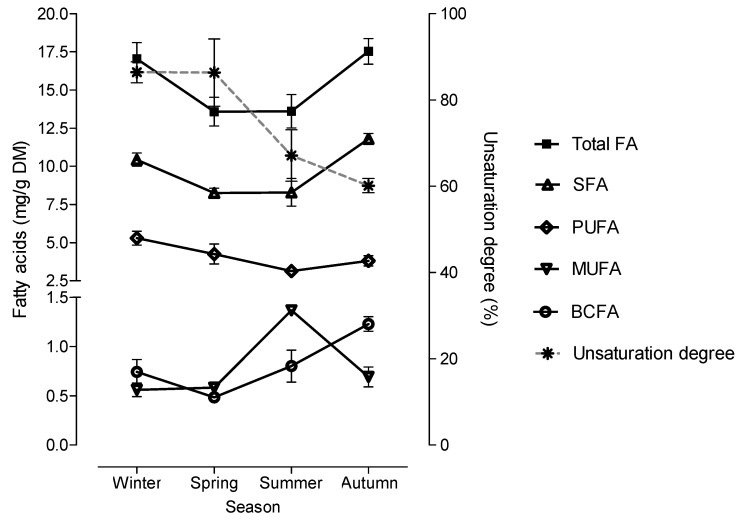
Variation in total fatty acids (FA), saturated fatty acids (SFA), polyunsaturated fatty acids (PUFA), monounsaturated fatty acids (MUFA), branched-chain fatty acids (BCFA) concentration and unsaturation degree in leaves according to season, unsaturation degree = (% monoenoic fatty acids × 1) + (% dienoic fatty acids × 2) + (% trienoic fatty acids × 3).

**Figure 5:**
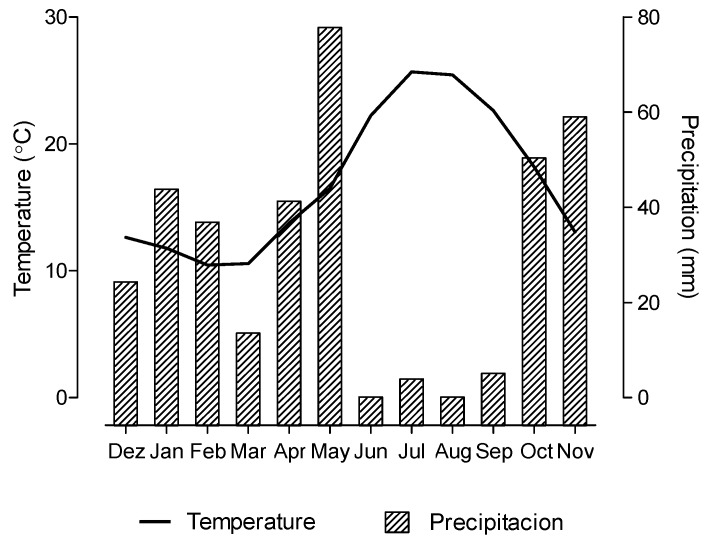
Environmental temperature and precipitation between December 2015 and November 2016.

The highest changes in FA composition of *C. ladanifer* morphological fractions throughout a year were observed in leaves, probably due to plant development stage and as a response to environmental conditions. The SFA, the major FA group in leaves, followed the variation of the total FA content throughout a year, as represented in Figure 4. Other groups of FA showed distinct behavior. During summer and autumn, the period which was observed higher environment temperatures (Figure 5), the sum of BCFA increased reaching the maximum value in autumn, while the PUFA content decreased. In agreement, the PCA showed that leaves from autumn was correlated with total and individual BCFA, and leaves collected during winter and spring had a higher association with total PUFA and 18:3n-3, the main PUFA present in leaves (Figure 3). The MUFA, composed exclusively by 18:1 *cis*-9, showed the highest value in summer when was reached the maximum environmental temperature and lower precipitation (Figure 5). The PCA also showed that leaves from summer was associated with MUFA content (Figure 3).

Fatty acids play multiple roles in plants as structural components of cell membranes and energy stores [42]. Moreover, FA are involved in cell signaling associated to plant development and response to abiotic and biotic stresses [43]. Re-modelling the cell membrane fluidity mediated by change in its FA composition is an adaptive response of plants to environmental stresses as low and high temperatures and drought as was already documented [44,45]. Plants respond to lower temperatures by increasing the levels of unsaturated FA, while an inverse relationship is observed at higher environmental temperatures [44,45]. Such changes in FA composition, which are mediated mainly by the activity of FA desaturases, allow the maintenance of the membrane fluidity at low and high environmental temperatures [45]. In the leaves of *C. ladanifer*, the change of unsaturation degree throughout the seasons is evident (Figure 5), with higher values during colder seasons (86.4% in winter and spring) than in summer (67.1%) and autumn (60.1%). Moreover, as described for other plant species, in *C. ladanifer* leaves the change in the degree of unsaturation with temperature variation results mainly from the change in the levels of trienoic FA [46,47]. In all *C. ladanifer* morphological fractions the only trienoic FA identified was the 18:3n-3, which varied significantly over the season in leaves, but also in stems. Although the FA composition in stems is more stable over seasons than in leaves (only two FA were changed in stems), in stems is also evident the reduction of 18:3n-3 in summer and autumn. Moreover, like in leaves, in stems the highest content 18:1 *cis*-9 was observed in summer. The increase in the MUFA content as plant response to higher environment temperatures was also found in other plant species [46].

Water-deficit stress also affects the plant lipid metabolism, leading to inhibition of lipid biosynthesis and stimulation of lipolytic and peroxidative activities [45]. Reduction of the 18:3n-3 in chloroplast monogalactosyldiacylglycerol and 18:2n-6 in phospholipid fractions was observed in drought-stressed rape (*Brassica napus*) plants [48].

Stress acclimating plants are able to adjust the membrane fluidity by changing levels of unsaturated FA [45], and the present results suggest that *C. ladanifer* have the ability to adapt to the seasonal changes of the Mediterranean climate throughout alteration of the leaves FA composition. Those alterations involve the replacement of the PUFA by FA with a lower unsaturation degree (MUFA) beyond the replacement by BCFA during hot seasons. However, if the increased content of SFA and MUFA with higher temperatures is well described for other plant species, the participation of BCFA in this adaptative mechanism is now known. To the best of our knowledge, only Randunz et al. [49] looking to the phospholipid composition of higher plants suggested that BCFA can replace PUFA in the membrane phospholipids and participate in the regulation of plant membrane fluidity. 

## 4. Materials and Methods

### 4.1. Plant Material Sampling

*Cistus ladanifer* shrubs with 2-3 years old were harvested in Baixo Alentejo region, Ourique, Southern Portugal (37°44′25.9″N/8°21′27.6″W), in a low-density forest composed by holm oak (*Quercus rotundifolia* L.) and cork oak (*Quercus suber* L.), where *C. ladanifer* plants emerged in a spontaneous way. Six plants (without root) per month were randomly collected for 12 months, between December 2015 and November 2016, with about one month between each sampling. The climate of the Mediterranean region is characterized by warm to hot dry summers and mild wet winters. The temperature and precipitation values observed between December 2015 and November 2016 at the Weather Station (Castro Verde) nearest to the sampling location are shown in Figure 5.

**Figure 6:**
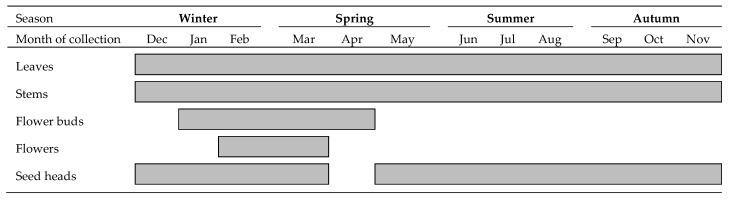
A sampling of several *C. ladanifer* morphological fractions throughout the year.

Samples were manually harvested with scissors and transported to the laboratory, where each plant was separated into leaves, stems, flower buds, flowers and seed heads. The presence of several morphological fractions throughout the year is present in Figure 6. Leaves and stems were collected in all months. Flower buds only occurred between January and May, and flowers in February and March. Only in April was not found seed heads in *C. ladanifer* plants. The flowers were only detected in six plants collected in February and March and so they were not enough to perform the analysis for each single month. So, a composite of those flowers was used for analysis.

After the separation of the morphological fractions, samples were dried at 40 °C with ventilation until constant weight. For each plant morphological fraction and month, the material from the six plants was pooled and ground in a mill with a sieve of 1 mm for analysis. 

### 4.2. Fatty Acid Analysis

Fatty acid methyl esters of each morphological part of *C. ladanifer* were prepared according to Guerreiro et al. [26]. Briefly, 1 mL of internal standard (19:0, 1 mg/mL in hexane) and 1 mL of toluene were added to 250 mg of sample, followed by the addition of 3 mL of 10% HCl solution in methanol (prepared by the addition of acetyl chloride to the methanol). After homogenization on vortex at slow speed, samples were maintained for 2 h at 70 °C in a water bath. Thereafter, the solution was left to cool at room temperature and subsequently neutralized with 5 mL of 6% potassium carbonate. Fatty acid methyl esters were extracted with 2 mL of hexane and dried over 0.5 g of anhydrous sodium sulfate. Finally, samples were centrifuged for 5 min at 350 g, the supernatant was transferred to new tubes and the solvent removed under nitrogen at 37 °C. The final residue was dissolved in 1.5 mL of hexane and stored at −20 °C until gas chromatography (GC) analysis.

Fatty acid methyl esters were analyzed by GC with flame ionization detection (GC-FID) using a Shimadzu GC-2010 Plus chromatograph (Shimadzu, Kyoto, Japan) equipped with a SP-2560 (100 m, 0.25 mm i.d., 0.20 µm film thickness, Supelco, Bellefonte, PA, USA) capillary column. Helium was used as carrier gas at the constant flow of 1 mL/min, and the injector and detector temperatures were 250 and 280 °C, respectively. The split ratio was 1:50 and the injection volume was 1 µL. Column oven programmed temperature were as follow: initial oven temperature of 50 °C was held for 1 min, increased to 150 °C at 50 °C/min and held for 20 min, then increased to 190 °C at 1 °C/min, and finally increased to 220 °C at 2 °C/min and maintained for 40 min. Identification of FA methyl esters was achieved by electron impact mass spectrometry using a Shimadzu GC-MS QP2010 Plus (Shimadzu, Kyoto, Japan) equipped with a SP-2560 capillary column (100 m, 0.25 mm i.d., 0.20 µm, film thickness, Supelco Inc., Bellefont, PA, USA). The gas chromatographic conditions were similar to the GC-FID conditions. The mass spectrometer conditions were as follows: ion source temperature, 200 °C; interface temperature, 220 °C; ionization energy, 70 eV; scan, 50–500 atomic mass units.

### 4.3. Statistical Analysis

Fatty acid content and composition for each plant morphological fraction was analyzed using a Proc MIXED option of SAS (SAS Institute Inc., Cary, NC, USA). Samples were grouped per season, considering three collections in each season (three repetitions per season). So, data for leaves, stems and seed heads were analyzed using the four seasons—winter, spring, summer and autumn, while for flower buds only was considered winter and spring. Flowers used for FA characterization were collected only once, during spring. Considering the sampling date, the months of December, January and February are part of the winter season; the March, April and May are part of spring season; the June, July, and August are part of the summer season; and September, October and November are part of the autumn season. Principal component analysis (PCA) was performed using a Statistica (TIBCO Statistica Academic version 13.3).

## 5. Conclusions

This study presents for the first time the FA content and composition of *C. ladanifer* morphological fractions (leaves, stems, flower buds, flowers and seed heads) and its alterations throughout a full year. Only leaves and stems were present all year and the interesting result was the presence of BCFA, i.e., iso-19:0 and iso-21:0, almost exclusively in leaves. Nevertheless, the highest FA content was observed in seed heads, which contained both seeds and the external structure. Seed heads and flowers presented the highest content of PUFA while leaves and stems presented the highest content of SFA. So, considering the FA content and composition is possible to discriminate the *C. ladanifer* morphological fractions, allowing identify three groups—leaves, stems and reproductive organs (flower buds, flowers and seed heads). In addition, leaves showed a great variation on FA composition throughout seasons, with the replacement of the PUFA by MUFA and BCFA during hot seasons, suggesting an adaptive response to environmental conditions. Conversely, the FA composition of the other morphological fractions is more stable over seasons, suggesting that leaves are more prone to stress adaptations due to environmental changes. 

## Figures and Tables

**Table 1:** Effect of season on fatty acid composition (mg/g DM) of *C. ladanifer* leaves.

	Winter	Spring	Summer	Autumn	SEM	*p* Values
Total FA	17.0^b^	13.6^a^	13.6^a^	17.5^b^	0.99	0.037
12:0	0.12	0.10	0.03	0.14	0.026	0.091
14:0	0.64^b^	0.64^b^	0.29^a^	0.55^b^	0.064	0.015
15:0	-	0.003	-	0.007	0.0024	0.219
16:0	2.22	2.01	1.82	1.89	0.102	0.103
16:1	-	0.003	-	0.007	0.0037	0.561
17:0	0.013	0.033	0.007	0.033	0.0089	0.140
17:1	0.007	0.027	0.007	0.010	0.0112	0.561
18:0	1.18^a^	1.08^a^	1.07^a^	1.73^b^	0.088	0.002
iso-19:0	0.28^a^	0.20^a^	0.31^a^	0.50^b^	0.040	0.004
18:1 *cis*-9	0.55^a^	0.55^a^	1.36^b^	0.68^a^	0.064	<0.001
18:2n-6	1.67	1.50	1.80	1.57	0.231	0.805
20:0	4.89^bc^	3.31^a^	3.89^ab^	5.97^c^	0.346	0.003
18:3n-3	3.62^c^	2.76^bc^	1.34^a^	2.24^ab^	0.310	0.005
iso-21:0	0.46^a^	0.29^a^	0.49^ab^	0.73^b^	0.077	0.024
21:0	0.06	0.10	0.08	0.11	0.034	0.794
22:0	0.95	0.70	0.74	0.92	0.070	0.086
24:0	0.38	0.27	0.35	0.46	0.045	0.124
Partial sums						
n-SFA	10.4^b^	8.26^a^	8.29^a^	12.0^b^	0.558	0.005
BCFA	0.74^a^	0.49^a^	0.80^a^	1.23^b^	0.110	0.009
MUFA	0.56^a^	0.58^a^	1.37^b^	0.69^a^	0.067	<0.001
PUFA	5.29^b^	4.26^ab^	3.15^a^	3.81^a^	0.434	0.044

FA—fatty acids; n-SFA—saturated fatty acids with linear chain; BCFA—branched chain fatty acids; PUFA—polyunsaturated fatty acids; SEM—standard error of the mean; Means with different letters within the same row are statistically different (*p* < 0.05).

**Table 2:** Effect of season on fatty acid composition (mg/g DM) of *C. ladanifer* stems.

	Winter	Spring	Summer	Autumn	SEM	*p* Values
Total FA	3.60	3.18	3.71	3.35	0.433	0.818
12:0	0.013^a^	0.010^a^	0.022^b^	0.020^b^	0.0019	0.008
14:0	0.057	0.051	0.058	0.056	0.0054	0.771
15:0	0.007	0.008	0.007	0.006	0.0009	0.510
16:0	0.64	0.58	0.53	0.49	0.052	0.282
16:1	0.006	0.006	0.006	0.007	0.0011	0.952
17:0	0.018	0.019	0.021	0.019	0.001	0.234
18:0	0.23	0.20	0.20	0.25	0.029	0.495
iso-19:0	0.002	0.005	0.004	0.009	0.0026	0.284
18:1 *cis*-9	0.25^a^	0.30^ab^	0.42^b^	0.25^a^	0.036	0.038
18:2n-6	0.49	0.43	0.39	0.35	0.066	0.514
20:0	0.69	0.52	0.62	0.64	0.133	0.828
20:1	0.015	0.021	0.018	0.024	0.0061	0.760
18:3n-3	0.11^b^	0.12^b^	0.07^a^	0.06^a^	0.012	0.011
21:0	0.030	0.025	0.029	0.027	0.0042	0.857
22:0	0.77	0.66	1.01	0.84	0.104	0.206
24:0	0.27	0.22	0.33	0.30	0.040	0.301
Partial sums						
n-SFA	2.73	2.29	2.82	2.65	0.335	0.702
MUFA	0.27	0.33	0.45	0.29	0.044	0.079
PUFA	0.60	0.56	0.45	0.41	0.077	0.322

FA—fatty acids; n-SFA—saturated fatty acids with a linear chain; BCFA—branched-chain fatty acids; PUFA—polyunsaturated fatty acids; SEM—standard error of the mean; Means with different letters within the same row are statistically different (*p* < 0.05).

**Table 3:** Effect of season on fatty acid composition (mg/g DM) of *C. ladanifer* flower buds and flowers.

	Flower Buds				Flowers
	Winter	Spring	SEM	*p* Values	Spring
Total FA	12.0	11.8	1.07	0.900	14.6
12:0	0.08	0.07	0.028	0.732	0.10
14:0	0.21	0.24	0.010	0.150	0.25
15:0	0.016	0.021	0.0025	0.293	0.033
16:0	2.71	2.92	0.204	0.551	4.07
16:1	0.011	0.008	0.0011	0.198	0.012
17:0	0.028	0.027	0.0072	0.931	0.025
18:0	0.51	0.57	0.107	0.704	0.26
iso-19:0	0.030	0.037	0.0111	0.679	0.021
18:1 *cis*-9	0.55	1.04	0.096	0.069	0.84
18:2n-6	3.47	3.90	0.147	0.174	5.41
20:0	1.69	1.13	0.271	0.280	0.87
20:1	0.19	0.076	0.0439	0.213	0.27
18:3n-3	1.82	1.32	0.229	0.265	1.83
21:0	0.056	0.040	0.0115	0.429	0.030
22:0	0.44	0.27	0.055	0.149	0.31
24:0	0.21	0.15	0.025	0.230	0.25
Partial sums					
n-SFA	5.95	5.42	0.710	0.653	6.20
MUFA	0.76	1.13	0.117	0.155	1.14
PUFA	5.28	5.22	0.376	0.912	7.24

FA—fatty acids; n-SFA—saturated fatty acids with linear chain; BCFA—branched-chain fatty acids; PUFA—polyunsaturated fatty acids; SEM—standard error of the mean.

**Table 4:** Effect of season on fatty acid composition (mg/g DM) of *C. ladanifer* seed heads.

	Winter	Spring	Summer	Autumn	SEM	*p* Values
Total FA	9.70	12.6	22.7	22.3	4.122	0.069
12:0	0.007	0.007	0.011	0.003	0.0022	0.089
14:0	0.04	0.07	0.10	0.06	0.019	0.098
15:0	0.008	0.009	0.004	0.006	0.0035	0.702
16:0	1.92	2.70	4.93	4.59	0.883	0.062
16:1	0.04	0.04	0.03	0.03	0.008	0.683
17:0	0.02	0.02	0.03	0.03	0.003	0.078
18:0	0.45	0.49	0.80	0.85	0.134	0.088
18:1 *cis*-9	1.55	1.38	1.99	2.06	0.297	0.272
18:2n-6	4.05	5.82	11.2	11.1	2.183	0.061
20:0	0.29	0.23	0.29	0.26	0.043	0.621
20:1	0.04	0.05	0.08	-	0.022	0.431
18:3n-3	1.02	1.60	3.08	3.13	0.623	0.054
21:0	0.02	0.02	0.02	0.02	0.003	0.911
22:0	0.14	0.12	0.13	0.12	0.015	0.622
24:0	0.10	0.08	0.08	0.08	0.010	0.362
Partial sums						
n-SFA	2.99	3.73	6.40	6.01	1.057	0.074
MUFA	1.64	1.48	2.10	2.10	0.310	0.330
PUFA	5.08	7.42	14.2	14.2	2.805	0.059

FA—fatty acids; n-SFA—saturated fatty acids with linear chain; BCFA—branched chain fatty acids; PUFA—polyunsaturated fatty acids; SEM—standard error of the mean.
